# RPLP2 activates TLR4 in an autocrine manner and promotes HIF-1α-induced metabolic reprogramming in hepatocellular carcinoma

**DOI:** 10.1038/s41420-023-01719-0

**Published:** 2023-12-05

**Authors:** Qingqing Yang, Xiangrui Meng, Jin Chen, Xiangsu Li, Yang Huang, Xueyi Xiao, Rongqing Li, Xudong Wu

**Affiliations:** 1 Department of Gastroenterology, The Yancheng Clinical College of Xuzhou Medical University, 224006 Yancheng, Jiangsu China; 2 Yancheng Medical Research Center of Nanjing University Medical School, Yancheng First Hospital, Affiliated Hospital of Nanjing University Medical School, The First People’s Hospital of Yancheng, 224006 Yancheng, Jiangsu China; 3 Department of Medical Genetics and Prenatal Diagnosis, The Affiliated Taizhou People’s Hospital of Nanjing Medical University, 225399 Taizhou, Jiangsu China

**Keywords:** Cancer metabolism, Mechanisms of disease, Glycobiology, Oncogenes, Extracellular signalling molecules

## Abstract

Metabolic reprogramming is a major feature of cancer, and aerobic glycolysis is one of the most widely studied metabolic reprogramming processes. Acidic ribosome protein P2 (RPLP2) is associated with both tumorigenesis and endoplasmic reticulum stress. However, limited knowledge exists regarding the role of RPLP2 in hepatocellular carcinoma (HCC) progression. In the present study, we observed a significant upregulation of RPLP2 in HCC tissues. Moreover, RPLP2 expression is closely correlated with patient prognosis and survival. The subsequent experimental validation demonstrated that RPLP2 exerted a regulatory effect on the expression of glycolytic enzymes and lactate production, thereby facilitating HCC cell proliferation. Mechanistically, the PI3K/AKT signalling pathway was found to play an important role in the regulation of hypoxia-inducible factor-1α (HIF-1α)-mediated aerobic glycolysis and cell growth. RPLP2 activates TLR4 on the surface of HCC cells and the downstream PI3K/AKT pathway through autocrine signalling. This activation then facilitates the entry of HIF-1α into the nucleus, enabling it to fulfil its transcriptional function. In conclusion, our findings suggested that RPLP2 induces a metabolic shift towards aerobic glycolysis and facilitates the progression of HCC through TLR4-dependent activation of the PI3K/AKT/HIF-1α pathway. Our study revealed the novel mechanism by which the ribosomal protein RPLP2 regulates glycolysis to promote HCC progression. These findings may offer a potential therapeutic target for HCC treatment.

## Introduction

HCC ranks fourth among the most lethal cancers [1] due to its high metastasis and recurrence rates, reflecting the poor efficacy of current therapeutic strategies [2]. Metabolic syndrome has been increasingly recognised as an important risk factor for HCC [3]. Metabolic alterations in neoplastic hepatocytes enhance their survival and growth capabilities under challenging conditions, modulate the tumour microenvironment, and compromise immune surveillance [4]. Therefore, conducting a comprehensive investigation into the metabolic mechanisms of HCC can facilitate the identification of pivotal biomarkers and the development of efficacious therapeutic strategies [5].

An important aspect of metabolic reprogramming is the reduction of oxidative phosphorylation (OXPHOS) within the tumour. To compensate, tumour cells produce energy through the aerobic glycolytic pathway, even in the presence of abundant oxygen in the cell [6]. This phenomenon is commonly referred to as the Warburg effect. Aerobic glycolysis was first discovered in rat liver carcinoma by Warburg and his colleagues and serves as a hallmark of liver cancer [7]. Aerobic glycolysis plays a crucial role in regulating glucose consumption and lactate production [8]. HCC cells promote cellular proliferation primarily by augmenting glucose uptake and driving energy production via the aerobic glycolytic pathway [9]. HIF-1α is a transcription factor closely related to oxygen homeostasis and aerobic glycolysis [10]. HIF-1α upregulates the expression of glucose transporters and glycolytic enzymes, including LDHA. This upregulation leads to an increase in glucose uptake, conversion to lactate, and extrusion of lactate from the cell [11]. Although it is termed the hypoxia-inducible factor, HIF-1α levels can be regulated by a range of factors, including COX-2, IGF2, ERBB2, EGFR, PI3K, HSP90, microtubule status, thioredoxin proteins and HDACs [12]. Moreover, HIF-1α is activated by various stimuli, even under normoxic conditions, and plays an important role in the metabolic reprogramming of cancer cells [13, 14].

The acidic ribosomal P protein family is present in eukaryotic cells and is specifically overexpressed in various types of cancer [15], and its dysregulation affects ribosome biogenesis, which is closely related to proliferation, glycolysis, autophagy, apoptosis and chemoresistance [16–19]. RPLP2, a member of this family, forms a dimer with RPLP1 and then combines with RPLP0 to form a ribosomal stalk [20], which together with the conserved structural domains of 28 S rRNA forms a GTPase-associated site [21] that promotes protein synthesis by recruiting translation factors and regulating the elongation phase of protein synthesis [22]. RPLP2 is highly expressed in gynaecological tumours, colon cancer, and lung adenocarcinoma [23–25], and a lack of RPLP2 leads to the accumulation of reactive oxygen species (ROS) and activation of the MAPK1/ERK2 signalling pathway, which induces cancer cell cycle arrest and autophagy [18]. Previous studies have demonstrated that RPLP2 functions as a pattern-associated molecular pattern (PAMP) that extracellularly binds to TLR4 on dendritic cell surfaces, thereby inducing dendritic cell maturation and activation in a TLR4-dependent manner and ultimately exerting tumour-immune effects [26]. However, the relationship between RPLP2 secretion and HCC progression remains unclear.

In this study, we identified RPLP2 as a tumour autocrine factor that is highly expressed in HCC and promotes glycolysis in HCC cells, thereby facilitating cell proliferation both in vivo and in vitro. Mechanistically, RPLP2 interacts with TLR4 on the surface of HCC cells, leading to activation of the PI3K/AKT signalling pathway. Subsequently, it targets HIF-1α and facilitates its translocation to the nucleus, where it exerts its transcriptional functions and upregulates the expression of glycolytic enzymes and glucose transport proteins associated with HCC progression. This investigation investigates the mechanism of RPLP2 in HCC, potentially offering a novel target for gene therapy against HCC.

## Results

### RPLP2 is overexpressed in HCC tissues and is secreted into the extracellular space

**Fig. 1:**
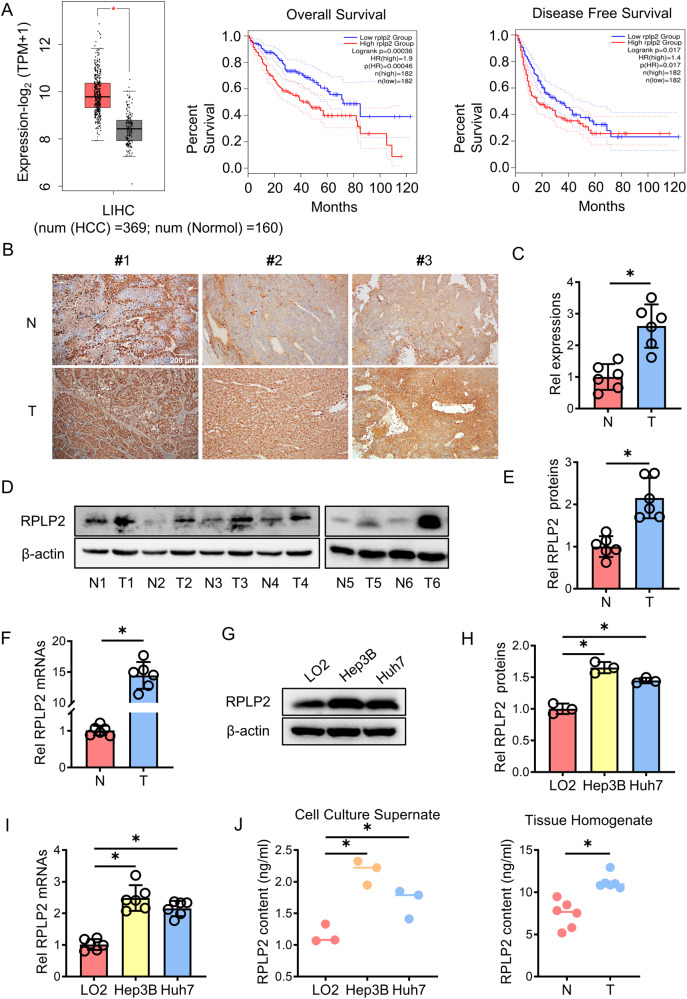
RPLP2 was overexpressed in the tissues of patients with HCC. **A** A TCGA dataset was used to determine the expression of RPLP2 in HCC tissue (*n* = 369) and nontumor hepatic tissue (*n* = 160) and its impact on patient prognosis, *P* < 0.05. **B**, **C** IHC detection of RPLP2 expression in HCC and adjacent normal tissues and the quantitative analysis (*n* = 10, mean ± SD). **D**, **E** RPLP2 protein expression levels in tumour and adjacent normal tissues of different patients were detected by Western blotting and the greyscale analysis (*n* = 6, mean ± SD). **F** Differential mRNA expression of RPLP2 in tumour and adjacent normal tissues measured by RT‒qPCR (*n* = 6, mean ± SD). **G**, **H** RPLP2 protein expression levels in LO2, Hep3B, and Huh7 cells were detected by Western blotting and the greyscale analysis (*n* = 3, mean ± SD). **I** The differential mRNA expression of RPLP2 between the normal liver cell line LO2 and two HCC cell lines, Hep3B and Huh7 measured by RT‒qPCR (*n* = 6, mean ± SD). **J** RPLP2 levels in cell culture supernatants (*n* = 3, mean ± SD) and tissue homogenate (*n* = 6, mean ± SD) measured by ELISA. Scale bar, 200 μm. *P < 0.05 versus corresponding control.

To investigate the role of RPLP2 in liver cancer pathogenesis, we examined its expression in liver cancer tissue using data from The Cancer Genome Atlas (TCGA) database. According to TCGA, RPLP2 is significantly upregulated in HCC tissue (*n* = 369) compared to nontumor hepatic tissue (*n* = 160), and its overexpression is associated with poor liver cancer patient prognosis, as indicated by the low overall survival rate and disease-free survival rate among those with high RPLP2 expression (Fig. 1A). In addition, we performed immunohistochemical analysis on HCC tissue and adjacent normal tissue from HCC patients, which confirmed the upregulated expression of RPLP2 specifically in HCC tissue (Fig. 1B, C). Furthermore, RNA and protein extraction from both HCC tissue and adjacent normal tissue further supported the finding that RPLP2 was highly expressed in HCC tissue compared to normal tissue (Fig. 1D–F).

Then, the normal liver cell line LO2 and two other HCC cell lines, Hep3B and Huh7, were utilised for RNA and protein extraction. The results revealed that the expression levels of RPLP2 were higher in Hep3B and Huh7 cells than in LO2 cells (Fig. 1G-I). Subsequently, tissue homogenates from both HCC tissue and adjacent normal tissue were obtained, along with cell supernatants collected from LO2, Hep3B and Huh7 cultures after 72 h for RPLP2 ELISA analysis. These findings demonstrated that liver cancer cells exhibit elevated expression levels of RPLP2. Moreover, the secretion levels of RPLP2 were higher in liver cancer cells, suggesting that it can have far-reaching effects after secretion into the extracellular space (Fig. 1J).

### RPLP2 knockdown suppresses the proliferation of HCC cells

**Fig. 2:**
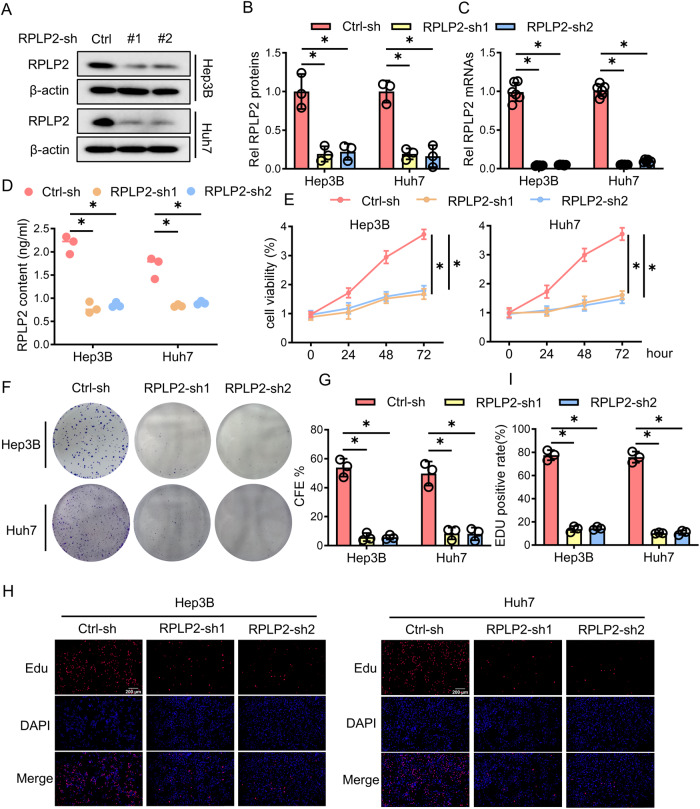
Knockdown of RPLP2 suppresses the proliferation of HCC cells. **A**, **B** After treating Hep3B and Huh7 cells with lentivirus, Western blotting was used to detect the protein expression level of RPLP2-sh, and greyscale analysis was performed (*n* = 3, mean ± SD). **C** RT‒qPCR was used to verify the transfection efficiency of RPLP2-sh (*n* = 6, mean ± SD). **D** Changes in RPLP2 levels in cell culture supernatant after knocking down RPLP2 (*n* = 3, mean ± SD). **E** CCK-8 assay was used to determine the changes in HCC cell viability after RPLP2 knockdown (*n* = 3, mean ± SD). **F**, **G** Changes in the colony-forming ability of HCC cells after knocking down RPLP2 and the quantitative analysis (*n* = 3, mean ± SD). **H**, **I** DNA synthesis ability in HCC cells after RPLP2 knockdown was investigated using an EdU assay and the quantitative analysis (*n* = 3, mean ± SD). Scale bar, 200 μm. *P < 0.05 versus corresponding control.

To examine the impact of RPLP2 on liver cancer cell proliferation, shRNA was employed to knock down RPLP2 in Hep3B and Huh7 cells. The knockdown efficiency was validated through reverse-transcriptase PCR and western blotting (Fig. 2A–C). Subsequently, a significant reduction in the extracellular secretion of RPLP2 by Hep3B and Huh7 cells was observed after knocking down RPLP2 (Fig. 2D). Cell viability assessed by CCK-8 assay demonstrated that silencing RPLP2 inhibited the proliferative capacity of HCC cells (Fig. 2E). Furthermore, colony formation assays revealed a substantial decrease in colony numbers in HCC cells with silenced RPLP2 compared to control cells (Fig. 2F, G). EdU staining revealed a marked decline in DNA synthesis within HCC cells following RPLP2 knockdown (Fig. 2H, I). Collectively, these experiments provide compelling evidence that RPLP2 can regulate HCC cell proliferation and that its depletion exerts anticancer effects.

### RPLP2 promotes glycolysis by regulating the expression and nuclear import of HIF-1α

**Fig. 3:**
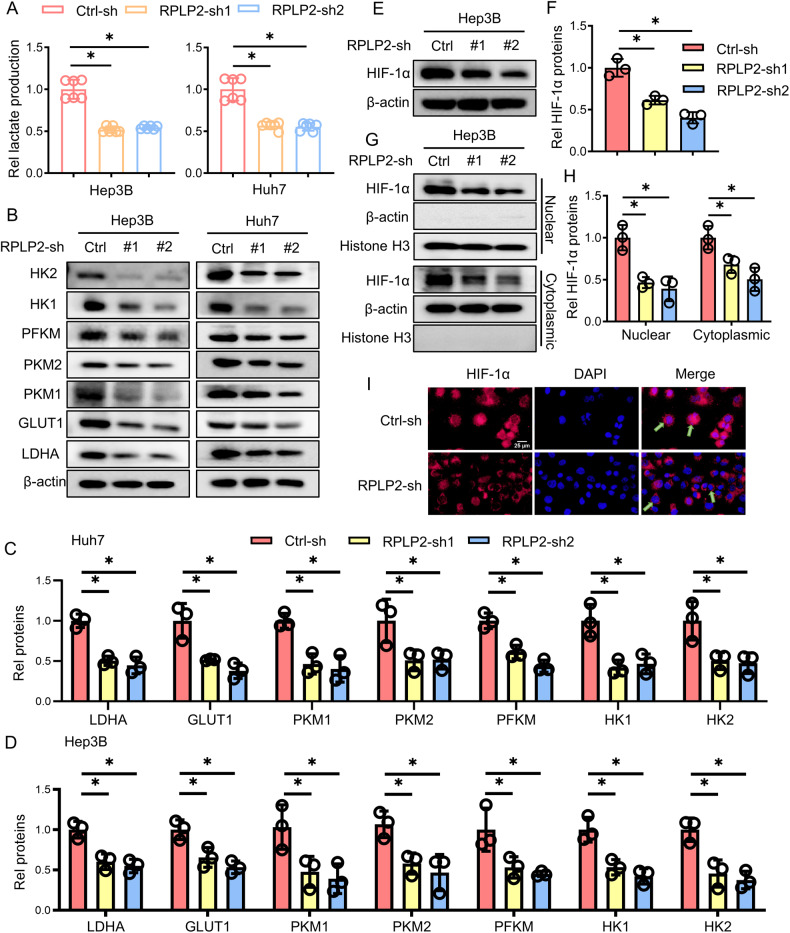
RPLP2 promotes aerobic glycolysis in HCC cells. **A** Lactate production at 24 h in HCC cells after RPLP2 knockdown (*n* = 3, mean ± SD). **B** Western blotting was used to detect the expression of glycolysis-related enzymes and glucose transporters, and **C**, **D** show the greyscale analysis (*n* = 3, mean ± SD). **E**, **F** Protein expression levels of HIF-1α after RPLP2 knockdown and the greyscale analysis (*n* = 3, mean ± SD). **G**, **H** Effect of RPLP2 on HIF-1α levels in the cytoplasm and nucleus (*n* = 3, mean ± SD). Knocking down RPLP2 decreased HIF-1α levels in the cytoplasm and nucleus. **I** Immunostaining of Hep3B cells with antibodies against HIF-1α (red). Knocking down RPLP2 decreased HIF-1α levels in the nucleus. Scale bar, 25 μm. *P < 0.05 versus corresponding control.

Aerobic glycolysis is recognised as a hallmark of cancer and plays a pivotal role in the proliferation of cancer cells [27]. However, it remains unclear whether RPLP2 can regulate aerobic glycolysis in HCC cells. To address this question, we compared lactate production between HCC cells and RPLP2 knockdown HCC cell lines, which revealed that knocking down RPLP2 significantly reduced lactate production in Hep3B and Huh7 cells (Fig. 3A). Furthermore, we found that silencing RPLP2 downregulated expression of key aerobic glycolysis proteins, such as LDHA, GLUT1, PKM1/2, PFKM and HK1/2, in Hep3B and Huh7 cells (Fig. 3B–D). Given that HIF-1α is a crucial transcriptional regulator of glycolytic enzyme expression under hypoxic conditions [10], we further examined its expression level after knocking down RPLP2. Our results showed that silencing RPLP2 resulted in decreased levels of both total (Fig. 3E, F) and nucleus-localised HIF-1α protein (Fig. 3G–I), suggesting that RPLP2 may modulate aerobic glycolysis by regulating the expression and nuclear translocation of HIF-1α.

**Fig. 4:**
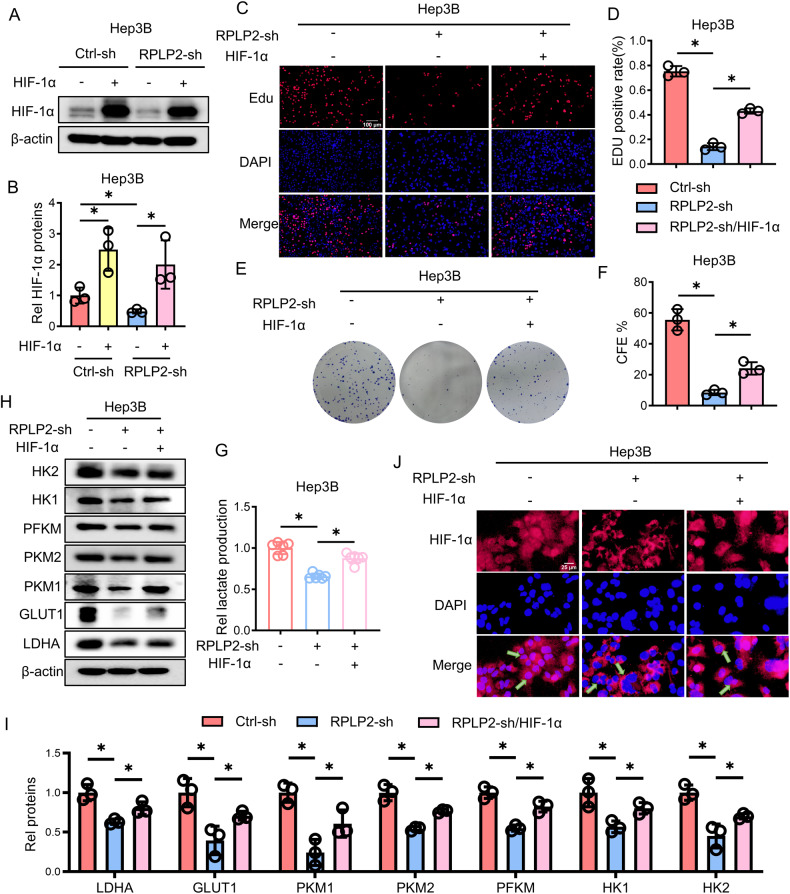
RPLP2 regulates the expression and nuclear uptake of HIF-1α. **A**, **B** Western blot analysis verifying the efficiency of HIF-1α overexpression in Hep3B cells and the greyscale analysis (*n* = 3, mean ± SD). **C**, **D** DNA synthesis was determined by EdU staining in Hep3B cells and the quantitative analysis. Overexpression of HIF-1α can reverse DNA synthesis in Hep3B cells inhibited by RPLP2 knockdown (*n* = 3, mean ± SD). Scale bar, 100 μm. **E** Colony-forming ability of Hep3B was rescued by HIF-1α overexpression and the quantitative analysis (*n* = 3, mean ± SD) is shown in (**F**). **G** HIF-1a overexpression increased lactate production. Cells with HIF-1α overexpression exhibited significantly increased lactate production compared with that of cells with RPLP2 knockdown at 24 h (*n* = 3, mean ± SD). **H**, **I** Western blotting and quantitative analysis were used to determine the changes in glycolysis-related enzymes and glucose transporters after overexpression of HIF-1α (*n* = 3, mean ± SD). **J** Immunostaining of Hep3B cells with antibodies against HIF-1α (red). Knocking down RPLP2 led to a decrease in the nuclear expression of HIF-1α, which was reversed by HIF-1α overexpression. Scale bar, 25 μm. *P < 0.05 versus corresponding control.

To elucidate the mechanism by which RPLP2 influences aerobic glycolysis in hepatoma cells and subsequently regulates proliferation, we employed lentivirus-mediated overexpression of HIF-1α (HIF-1α-OE) in Hep3B cells (Fig. 4A, B). EdU staining revealed that the knockdown of RPLP2 significantly attenuated DNA synthesis in Hep3B cells; however, upon overexpression of HIF-1α, DNA synthesis exhibited a significant reversal (Fig. 4C, D). Cloning experiments demonstrated that ectopic expression of HIF-1α effectively rescued the colony-forming ability of Hep3B cells impaired by RPLP2 knockdown (Fig. 4E, F). Collectively, these findings indicate that overexpression of HIF-1α can restore the proliferative capacity of liver cancer cells suppressed by RPLP2 depletion. Furthermore, we observed that lactate production by Hep3B cells was increased following HIF-1α overexpression after RPLP2 knockdown (Fig. 4G). In addition, the nuclear localisation and expression levels of key aerobic glycolysis-related proteins were significantly restored upon overexpression of HIF-1α (Fig. 4H–J). Altogether, these results demonstrate that aerobic glycolysis is compromised upon RPLP2 knockdown but can be rescued by the exogenous expression of HIF-1α.

### The PI3K/AKT pathway is involved in the regulation of HIF-1α by RPLP2

**Fig. 5:**
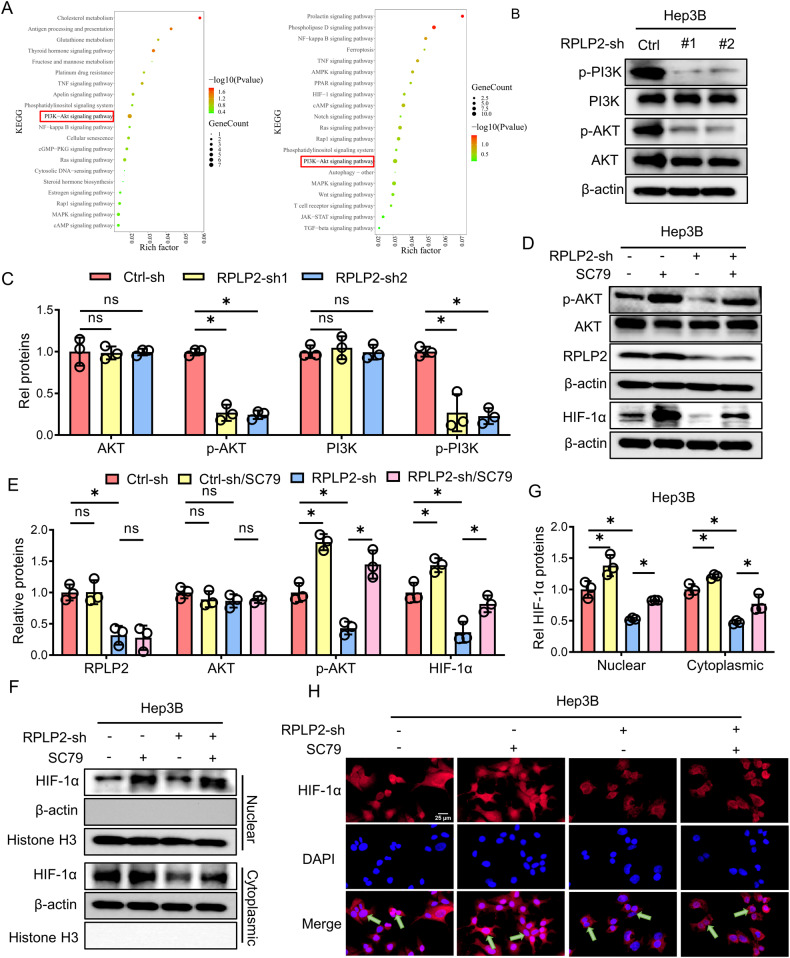
RPLP2 affects HIF-1α by regulating the PI3K/AKT pathway. **A** KEGG pathway analysis of differentially expressed genes when RPLP2 was knocked down in Hep3B and Huh7 cells. **B** Western blot analysis showed a decrease in the protein levels of p-PI3K and p-AKT after RPLP2 knockdown and the quantitative analysis (*n* = 3, mean ± SD) is shown in (**C**). **D** After treatment of Hep3B cells with the AKT activator SC79 (10 µM) for 24 h, Western blotting was used to detect the expression of HIF-1α and p-AKT, and quantitative analysis (*n* = 3, mean ± SD) was performed in (**E**). After treatment with SC79, the expression of HIF-1α and p-AKT was rescued. **F** Western blotting was used to determine the effect of SC79 on HIF-1α levels in the cytoplasm and nucleus, and the quantitative analysis (*n* = 3, mean ± SD) was performed in (**G**). SC79 restored HIF-1α levels in the cytoplasm and nucleus. **H** Immunostaining of Hep3B cells with antibodies against HIF-1α (red). Knocking down RPLP2 led to a decrease in the nuclear expression level of HIF-1α, which was abrogated by SC79. Scale bar, 25 μm. *P < 0.05 versus corresponding control; ns means nonsignificant.

The present study investigates the underlying mechanism by which RPLP2 modulates the nuclear translocation of HIF-1α. RNA-seq analysis revealed significant alterations in the PI3K/AKT pathway upon knockdown of RPLP2 in both Hep3B and Huh7 cell lines (Fig. 5A). Subsequently, we observed a reduction in p-PI3K and p-AKT levels following RPLP2 knockdown specifically in Hep3B cells (Fig. 5B, C). It has been well-established that the PI3K/AKT pathway can regulate HIF-1α expression, thereby influencing glycolysis [28]. To confirm that RPLP2 regulates HIF-1α expression and nuclear translocation by modulating the PI3K/AKT pathway, we treated Hep3B cells with SC79, an AKT activator known to enhance AKT phosphorylation. Remarkably, SC79 treatment rescued the downregulated expression of HIF-1α and p-AKT caused by RPLP2 knockdown in Hep3B cells (Fig. 5D, E) while also reversing its impaired nuclear entry (Fig. 5F–H). Collectively, these experiments provide compelling evidence supporting the role of RPLP2 in regulating HIF-1α via modulation of the PI3K/AKT pathway.

### RPLP2 activates the TLR4-mediated PI3K/AKT cascade through autocrine signalling

**Fig. 6:**
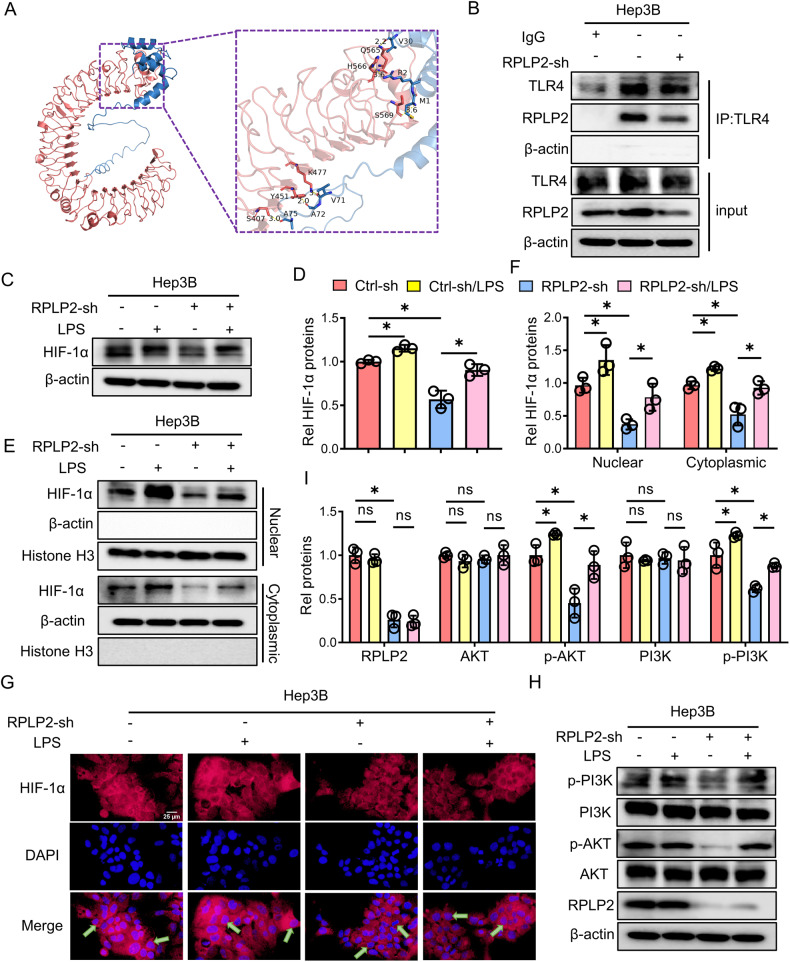
RPLP2 secreted into the extracellular space activates the TLR4/PI3K/AKT cascade. **A** Zdock software predicted the binding sites of RPLP2 and TLR4. **B** Co-IP validated that RPLP2 binds to TLR4 in Hep3B cells. **C**, **D** Hep3B cells were treated with the TLR4 activator LPS (100 ng/mL) for 24 h, and Western blot and quantitative analyses showed that the expression level of HIF-1α was increased in RPLP2 knockdown cells treated with LPS compared to untreated RPLP2 knockdown cells (*n* = 3, mean ± SD). **E**, **F** Western blotting was used to determine the effect of LPS on HIF-1α levels in the cytoplasm and nucleus and the quantitative analysis (*n* = 3, mean ± SD). LPS increased HIF-1α levels in the cytoplasm and nucleus. **G** Immunostaining of Hep3B cells with antibodies against HIF-1α (red). Knocking down RPLP2 led to a decrease in the nuclear expression level of HIF-1α, which was abrogated by LPS. **H**, **I** After treatment with LPS, western blotting detected that downstream p-PI3K and p-AKT both rotated compared to RPLP2 knockdown (*n* = 3, mean ± SD). Scale bar, 25 μm. *P < 0.05 versus corresponding control; ns means nonsignificant.

To gain further insight into the molecular mechanism by which RPLP2 regulates PI3K/AKT, we conducted a literature review and discovered that RPLP2 can bind to the dendritic cell surface receptor TLR4 [26], which mediates glycolysis and plays a crucial role in immune cells [29]. Therefore, we hypothesize that TLR4 is the link between RPLP2 and glycolysis. First, using Zdock software, we predicted the binding sites of RPLP2 and TLR4 (Fig. 6A), which were then confirmed through a Co-IP assay in Hep3B cells (Fig. 6B). We added LPS, a specific activated receptor for TLR4, to Hep3B cells with sh-RPLP2 knockdown and observed that total content (Fig. 6C, D) and nuclear number (Fig. 6E–G) of HIF-1α increased compared to those in untreated Hep3B cells with sh-RPLP2 knockdown. Subsequently, downstream p-PI3K and p-AKT were detected and found to have rotated (Fig. 6H, I). These results suggest that the autocrine binding of RPLP2 to the receptor TLR4 on HCC cell surfaces regulates the downstream PI3K/AKT pathway.

### RPLP2 regulates HIF-1a and promotes tumour growth in vivo

**Fig. 7:**
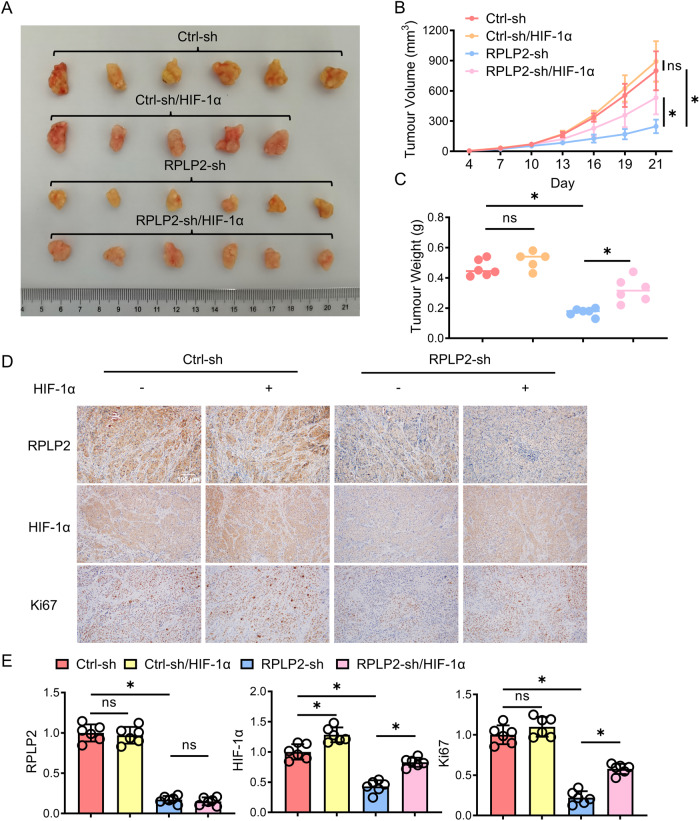
The RPLP2/HIF-1α axis modulates glycolysis and tumour proliferation in mice. **A** Cells were subcutaneously injected into the flanks of BALB/c nude mice to investigate proliferative ability. Images of the dissected tumours from the BALB/c nude mice (*n* = 6). **B** The tumour growth curves of each group are shown (*n* = 6, mean ± SD). **C** The tumour weight of each group is shown (*n* = 6, mean ± SD). **D**, **E** IHC staining of xenograft tumour sections with the indicated treatment and quantitative analysis (*n* = 6, mean ± SD). The scale bars represent 100 μm. *P < 0.05 versus corresponding control; ns means nonsignificant.

To investigate the role of the RPLP2-HIF-1α axis in HCC development, we subcutaneously injected Hep3B cells transfected with control-sh, HIF-1α-OE, RPLP2-sh and RPLP2-sh + HIF-1α-OE into each group of BALB/c nude mice. After 4 weeks, the mice were euthanized, and the size and weight of the subcutaneous tumours were measured. The xenograft animal model further validated that RPLP2 knockdown inhibited HCC proliferation, while overexpression of HIF-1α rescued this inhibition caused by sh-RPLP2 (Fig. 7A–C). Immunohistochemistry analysis revealed a significant reduction in Ki67 expression levels in the tumours formed by sh-RPLP2 HCC cells. However, upon overexpression of HIF-1α, Ki67 expression was restored (Fig. 7D, E). These findings suggest an indirect regulation of HCC proliferation through RPLP2-mediated modulation of HIF-1α.

## Discussion

**Fig. 8:**
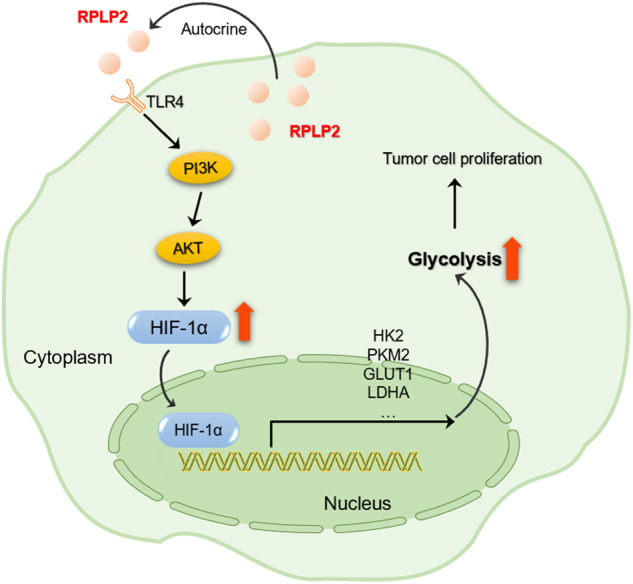
Proposed mechanism by which RPLP2 regulates glycolytic reprogramming and HCC progression. RPLP2 is secreted into the extracellular space and binds to the TLR4 receptor by autocrine signalling, activating the downstream PI3K/AKT cascade reaction and upregulating the expression of HIF-1α and its entry into the nucleus. HIF-1α increases the transcription level of glycolysis-related enzymes and glucose transporters, promoting glycolytic reprogramming and HCC progression.

Ribosomal proteins are emerging as potential targets for the molecular therapy of cancer, and in addition to their protein synthesis functions, their extra ribosomal functions are critical for tumour growth. Ribosome protein L52 mediates hypoxia-induced apoptosis resistance and metastasis in breast cancer [30]. Ribosomal protein L39 sustains mitochondrial cristae morphogenesis and facilitates reactive oxygen species production in ovarian cancer [31]. The ribosomal protein P0 significantly enhances the repair of DNA double-strand breaks through nonhomologous end joining, thereby playing a pivotal role in DNA repair and promoting tumour radioresistance [32]. However, there are currently no reports indicating the potential tumour autocrine function of the P protein family. In this study, we identified RPLP2 as a PAMP that governs aerobic glycolysis. Our findings suggest that RPLP2 activates downstream PI3K/AKT cascade reactions by directly binding to TLR4 in an autocrine manner. Importantly, RPLP2 regulates aerobic glycolysis and liver cancer cell proliferation via TLR4-mediated HIF-1α translocation into the nucleus. Therefore, the RPLP2/TLR4/PI3K/AKT axis provides a signalling pathway for regulating HIF-1α and subsequently promoting aerobic glycolysis and cancer cell growth (Fig. 8).

The ubiquitous observation of aerobic glycolysis in human cancers, its persistence even under normoxic conditions, and its correlation with tumour aggressiveness suggest that the glycolytic phenotype endows a considerable proliferative advantage to cancer cells during cancer somatic evolution and is indispensable [33]. Therefore, increased glycolysis is a fundamental characteristic of the malignant phenotype and serves as a hallmark of cancer. The pivotal role of aerobic glycolysis in cancer progression has been extensively investigated. Transcription factor homeobox A3 (HOXA3) orchestrates the transcriptional activation of aerobic glycolysis, thereby inducing a profound enhancement in glioblastoma cell proliferation and tumour growth [34]. FOXD1 upregulates GLUT1 expression, thereby facilitating the proliferation, invasion, and metastasis of pancreatic cancer cells through the regulation of aerobic glycolysis [35]. Let-7b-5p inhibits the growth and metastasis of breast tumours by inhibiting HK2-mediated aerobic glycolysis [36]. Therefore, regulating HCC glycolysis may be an effective strategy for treating HCC [37]. In this study, we identified a novel tumour autocrine molecule, RPLP2, which exerts regulatory control over the Warburg effect in HCC cells. The absence of RPLP2 results in reduced expression levels of aerobic glycolytic enzymes and glucose transporters, as well as decreased lactate production, indicating that RPLP2 drives metabolic reprogramming towards aerobic glycolysis. Our findings suggest that RPLP2 promotes HCC progression through the modulation of glucose metabolism.

Multiple cellular pathways have been proven to promote the reprogramming of glucose metabolism. P53 modulates the balance between the utilisation of respiratory and glycolytic pathways through cytochrome *c* oxidase 2 (SCO2) [38]. A myc-dependent global metabolic transcriptome drives metabolic reprogramming in activated, primary T lymphocytes [39]. HIF-1α-mediated induction of Pdk1 was found to regulate glucose oxidation by preventing the entry of pyruvate into the tricarboxylic cycle [40]. HIF-1α, as the most extensively studied aerobic glycolysis regulatory molecule, is overexpressed in many cancers and is the main transcription factor that initiates aerobic glycolysis [10], controlling cell metabolism to promote tumour growth. Therefore, blocking the function of HIF-1α may be beneficial for inhibiting cancer progression. We investigated the effect of RPLP2 on HIF-1α and found that in cells with RPLP2 knockdown, the nuclear level of HIF-1α was significantly reduced. Therefore, we speculate that RPLP2 regulates aerobic glycolysis by influencing the entry of HIF-1α into the nucleus.

A consequence of the many key genetic changes in cancer cells is the increased function of HIF-1α. Moreover, it is worth investigating how RPLP2, as a ribosomal protein, regulates HIF-1α. Previous studies have shown that RPLP2 can be secreted as a PAMP into the extracellular space and bind to TLR4 receptors on the surface of dendritic cells [26]. TLR4 is a Lipopolysaccharide receptor, which is mainly expressed in macrophages, dendritic cells and other immune cells [41]. However, it has been found that TLR4 is expressed in HCC cells [42] and is critical in DNA damage, cellular aging, and autophagy [43, 44]. Moreover, TLR4 plays an important role in autocrine signalling. The secretion of the HSP90 cochaperone protein Morgana induces cancer cell migration by activating TLR2, TLR4, and LRP1 [45]. Tumour-derived inflammatory glycoprotein PTX3 promotes melanoma cell invasion through a TLR4-dependent pathway [46]. Tumour-promoting protein PAUF secreted by cancer cells promotes the migration of human pancreatic cancer cells through TLR4/MyD88 signal [47]. TLR4 has been shown to be expressed in HCC cells, and our study suggests that RPLP2 can bind to TLR4 on the surface of HCC cells. PI3K/AKT is a downstream signalling pathway of TLR4 [48], and knockdown of RPLP2 resulted in the inhibition of PI3K/AKT, while the use of a TLR4 agonist reversed PI3K/AKT signalling inhibition, suggesting that RPLP2 activates the downstream PI3K/AKT pathway via TLR4. More importantly, HIF-1α is regulated by PI3K/AKT signalling [14], and RPLP2 deficiency inhibits HIF-1α entry into the nucleus, whereas AKT agonists can restore the nuclear level of HIF-1α. We demonstrated that RPLP2 regulates HIF-1α mainly through autocrine activation of the TLR4/PI3K/AKT cascade, which in turn affects HIF-1α entry into the nucleus. Therefore, the autocrine activation of TLR4 by RPLP2 is an important mechanism of glycolysis in HCC.

In this study, we demonstrated that the autocrine function of RPLP2 in the extracellular domain promotes aerobic glycolysis in HCC cells by activating TLR4 and ultimately affects liver cancer cell proliferation. However, a small molecule inhibitor of RPLP2 has not yet been developed, limiting its application in clinical research. Furthermore, the functional role of RPLP2 within tumour cells remains unknown, and whether it is involved in other cancer-promoting pathways requires further investigation.

In conclusion, our findings revealed a novel function of the ribosome protein RPLP2 as a regulator of aerobic glycolysis through its influence on HIF-1α. Moreover, our study demonstrated that RPLP2 interacts with TLR4 to promote cancer development. We elucidated the molecular mechanism by which RPLP2 influences metabolic reprogramming via autocrine signalling and provided evidence for its role in promoting HCC development both in vivo and in vitro. Therefore, targeting RPLP2 could be a potential therapeutic strategy for HCC.

## Materials and methods

### Database analysis

The differential expression of RPLP2 between HCC tissues and adjacent noncancerous tissues was analysed using Gene Expression Profiling Interactive Analysis (GEPIA, http://gepia.cancer-pku.cn/) from The Cancer Genome Atlas (TCGA, https://cancergenome.nih.gov/), followed by Kaplan‒Meier survival prediction analysis.

### Human HCC tissues

Sectioned HCC tissues (*n* = 10) were collected from the Pathology Department of Yancheng Clinical College of Xuzhou Medical University (Yancheng, China) between January 1, 2020, and December 30, 2022. HCC tissues and adjacent noncancerous tissues (*n* = 6) were obtained from patients who met the Barcelona Clinic Liver Cancer guidelines definition and underwent surgical treatment at the General Surgery department between January 1, 2023, and June 30, 2023; all patients provided informed consent. All specimens were promptly acquired after surgical resection, snap-frozen in liquid nitrogen, and stored at −80 °C. The study was approved by the Ethics Committee of Yancheng Clinical College of Xuzhou Medical University (No.: 2023-K-023).

### Cell cultures and reagents

The immortalised normal hepatocyte line LO2 and two types of HCC cells, Hep3B and Huh7, were procured from FuHeng Biology (Shanghai, China). LO2 and Huh7 cells were cultured in high glucose DMEM (Gibco, New York, USA), while Hep3B cells were maintained in MEM (Gibco, New York, USA) supplemented with 10% foetal bovine serum (FBS, Gibco, USA), 100 U/mL penicillin and 100 U/mL streptomycin at 37 °C with 5% CO_2_. The AKT activator and TLR4 activator were purchased from MedChemExpress (MCE, New Jersey, USA) and were separately dissolved in DMSO and H_2_O. These cell lines underwent authentication through short tandem repeat (STR) profiling to ensure their identity and were found to be free of mycoplasma contamination.

### Quantitative real-time PCR (RT‒qPCR)

Total RNA was extracted using TRIzol reagent (Vazyme, Nanjing, China) and quantified by measuring the absorbance at 260 nm. Subsequently, it was purified and reverse-transcribed into cDNA using a reverse transcription kit (Vazyme, Nanjing, China). RT‒qPCR analysis was performed using 2 × Taq Pro Universal SYBR qPCR Master Mix (Vazyme, Nanjing, China). The relative mRNA levels of RPLP2 were calculated using the 2–ΔΔCT method with β-actin serving as an internal reference. Primers for RPLP2 and β-actin were synthesised by Genscript Biotech Corporation (Cayman Islands, USA) and were as follows: β-actin: F: CTCCATCCTG-GCCTCGCTGT; R: GCTGTCACCTTCACCGTTCC; RPLP2: F: CCGGCTCAAC-AAGGTTATCAG; R: TTGCAGGGGAGCAGGAATTTA.

### Cell counting Kit-8 (CCK-8) assay

Cell viability was assessed using the CCK-8 assay. HCC cells were seeded into 96-well plates at a density of 3 × 10^3^ cells/well and incubated for 0, 24, 48, and 72 h in a controlled environment at 37 °C. Subsequently, each well was supplemented with 10 µL of CCK-8 reagent (Abbkine, Wuhan, China) and further incubated for an additional hour at the same temperature. The absorbance values of each experimental group were measured at a wavelength of 450 nm using a microplate reader (Thermo Fisher, Massachusetts, USA).

### Colony formation assay

The colony formation rate serves as an indicator of cellular proliferation capacity. Cells were seeded onto 6-cm plates at a density of 1000 cells per plate and cultured in complete medium for 10 days until colonies became visible to the naked eye. Subsequently, the plates were fixed with 4% polyoxymethylene and stained using crystal violet. After washing with tap water, they were photographed and quantified using ImageJ software.

### 5‑ethynyl‑2ʹ‑deoxyuridine (EdU) proliferation assay

The EdU proliferation assay was employed to quantify cell proliferation. Cells were seeded in 96-well plates at a density of 3 × 10^3^ cells/well for a duration of 24 h. Following incubation with Abbkine’s EdU solution (10 μM, Wuhan, China) for a period of 2 h, the cells were fixed using a solution containing 4% paraformaldehyde and permeabilized utilising PBS supplemented with 0.5% Triton X-100 for a duration of 10 min. Subsequently, the cells were subjected to Apollo staining solution and incubated for approximately half an hour. DAPI was used to stain nuclei. Fluorescence analysis was conducted on five randomly selected fields of view employing an Olympus microscope from Tokyo, Japan to assess proliferation rates. The nuclei appeared blue after staining with DAPI, while proliferating cells exhibited red fluorescence due to the presence of Apollo-bound EdU. The proportion of proliferating cells was determined based on these observations. The total cell count and proliferating cell count were quantified using ImageJ software.

### Western blot analysis

Total protein was lysed using RIPA buffer (Servicebio, Wuhan, China) supplemented with phosphatase inhibitor and protease inhibitor (Beyotime, Jiangsu, China). Additionally, nuclear and cytosolic proteins were extracted utilising the ExKineTM Nuclear and Cytoplasmic Protein Extraction Kit (Abbkine, Wuhan, China) following the manufacturer’s instructions. Subsequently, protein solutions were heated in 5 × loading buffer at 98 °C for 10 min and then separated on SDS‒PAGE gels. After being transferred onto PVDF membranes (Millipore, Massachusetts, USA), the protein contents were probed using the following antibodies: anti-RPLP2 (1:1000, ab154958, Abcam, Cambridge, UK), anti-HIF-1α (1:1000, A7684, ABclonal, Wuhan, China), anti-LDHA (1:1000, #3582 S), anti-GLUT1 (1:1000, #73015 S), anti-PKM1 (1:1000, #7067 S), anti-PKM2 (1:1000, #4053 S), anti-HK1 (1:1000, #2024 S), anti-HK2 (1:1000, #2867 S) (all from CST, Massachusetts, USA), anti-PI3K (1:1000, AF6242), anti-p-PI3K (1:500, AF3242)(all from Affinity, Jiangsu, China), anti-PFKM (1:1000, 55028-1-AP), anti-AKT (1:1000, 10176-2-AP), anti-p-AKT (1:500, 28731-1-AP), and anti-TLR4 (1:1000, 19811-1-AP) (all from Proteintech, Wuhan, China). Anti-histone H3 (1:1000, A2348, ABclonal, Wuhan, China) and anti-β-actin (1:50000, AC026, ABclonal, Wuhan, China) served as controls. HRP-conjugated goat anti-rabbit (AS063) and mouse IgG (AS064) (1:10000, ABclonal, Wuhan, China) were used as the secondary antibodies. Immunoblotting was visualised with ECL (ABclonal, Wuhan, China) using the ChemiDoc imaging system (Bio-Rad, California, USA), and protein bands were quantified with ImageJ.

### Construction of stable cells

The vector VP013 lentivirus carrying shRNA for RPLP2 was purchased from Tsingke Biotech (Beijing, China). HIF-1α-overexpression lentivirus was synthesised and provided by GenePharma (Suzhou, China). The specific sequences of RPLP2 shRNAs are as follows: RPLP2-shRNA1: GGAGTCTGAAGAGTCAGATGA; RPLP2-shRNA2: GGTTATC-AGTGAGCTGAATGG. The order of the components of the HIF-1α-overexpression lentivirus was Ubi-MCS-3FLAG-SV40-EGFP-IRES-puromycin. Exponentially growing Hep3B and Huh7 cells (~60–80% confluent) were transfected with the lentivirus and selected using 2 μg/ml puromycin. The level of knockdown or overexpression protein was evaluated through Western blot assays or RT‒qPCR.

### Immunofluorescence staining

Cells were seeded onto glass slides and fixed with 4% paraformaldehyde. Subsequently, they were permeabilized using Triton X-100 and incubated overnight at 4 °C with the anti-HIF-1α antibody (1:500, #79233 S, CST, Massachusetts, USA). Following this step, samples were incubated with Alexa Fluor 594-conjugated donkey anti-mouse IgG antibody (1:500, A24411, Abbkine, Wuhan, China), while cell nuclei were stained using DAPI from Sigma-Aldrich (Germany). Immunofluorescence was examined utilising a fluorescence microscope (Hitachi HT7700, Tokyo, Japan) and further digitally processed for contrast enhancement through Adobe Photoshop.

### Immunohistochemistry (IHC)

Paraffin-embedded and formalin-fixed tissue sections were deparaffinized and rehydrated, followed by blocking endogenous peroxidase activity using 3% hydrogen peroxide (H2O2). The sections underwent heat-mediated antigen retrieval with sodium citrate buffer (pH=6) in a pressure cooker and were then incubated overnight at 4 °C with the following antibodies: anti-RPLP2 (1:100, ab154958, Abcam, Cambridge, UK), anti-HIF-1α (1:100, 66730-1-Ig, Proteintech, Wuhan, China), and anti-Ki67 (1:50, ab16667, Abcam, Cambridge, UK). Subsequently, the cells were treated with the appropriate secondary antibody (1:1000, A21020, A21010, Abbkine, Wuhan, China) and stained using 3.3-diaminobenzidine tetrahydrochloride (DAB). After counterstaining with haematoxylin, the sections were sealed with neutral resins and observed under a microscope (Hitachi HT7700, Tokyo, Japan).

### Enzyme-linked immunosorbent assay (ELISA)

The levels of RPLP2 in the tissue homogenate and cell culture supernatant from different groups were quantified using a human RPLP2 ELISA kit (Jiangsu Meimian Industrial, China) following the manufacturer’s instructions. The absorbance values of each sample were measured at a wavelength of 450 nm using a microplate reader (Thermo Fisher, Massachusetts, USA).

### Protein–protein docking

The crystal structures of TLR4 (PDBID: 4G8A) were obtained from the Protein Database (PDB) website, while RPLP2 (PDBID: 4BEH) was acquired from the Universal Protein (UniProt) website. The removal of water molecules and small compounds was carried out, followed by hydrogen-bond optimisation to preserve the three-dimensional protein binding. Zdock software was utilised for protein‒protein docking, with TLR4 selected as the receptor and the RPLP2 structure chosen as the ligand. Docking parameters included protein-surface structure and surface potential.

### Coimmunoprecipitation (Co-IP) assay

Total lysate was extracted from Hep3B cells using NP-40 cell lysis buffer (Beyotime, Jiangsu, China) containing protease inhibitors. After centrifugation, the supernatant was subjected to SDS‒PAGE and subsequent immunoblotting. To examine the endogenous TLR4-RPLP2 interaction, the cell lysates were incubated overnight at 4 °C with an anti-TLR4 antibody (4 μg/ml, 66350-1-Ig, Proteintech, Wuhan, China) or control IgG (1:100, AC011, ABclonal, Wuhan, China). Then, the lysates were incubated with protein G/A beads (Invitrogen, Carlsbad, CA, USA) for 3 h at 4 °C. The beads were carefully washed with precooled PBS and mixed with protein loading buffer before being boiled and analysed by immunoblotting using the anti-TLR4 antibody (1:1000; 19811-1-AP, Proteintech, Wuhan, China) and anti-RPLP2 antibody (1:1000; ab154958, Abcam, Cambridge, UK).

### Lactate-level measurements

Lactate levels were measured using the Lactate Assay Kit (Abbkine, Wuhan, China). Specifically, lactate reacted with an enzyme mix to generate a product. Subsequently, a lactate probe was added to produce colour, and the absorbance was read at a wavelength of 570 nm using a microplate reader (Thermo Fisher, Massachusetts, USA).

### Xenograft models

The 6-week-old male BALB/c nude mice were randomly divided into four groups (six per group), and injected with ~1 × 10^6^ cells at upper backs. Tumour size was measured on a weekly basis, and one month later, the BALB/c nude mice were euthanized for tumour size assessment (including mice that died during observation). All animal procedures were conducted in accordance with the guidelines (released by the Ministry of Science and Technology of the People’s Republic of China on September 30, 2006) for animal welfare after obtaining approval from the medical laboratory animal ethics committee of Jiangsu Medical Vocational College (No.: SYLL-2023-701).

### Statistical analysis

Each experiment was independently performed with a minimum of three replicates, and the results are expressed as the mean ± standard deviation (SD). Statistical analyses were conducted using GraphPad Prism 9 software (GraphPad Software, CA, USA). Student’s *t* test was employed for comparisons between two groups, while one-way or two-way analysis of variance (ANOVA) was used for three or more samples. Differences were considered statistically significant when *P* < 0.05 and denoted with an asterisk (*).

### Supplementary information

WB Original image

## Data Availability

All data generated or analysed in this study are included in the article and its supplementary files.
