# Supplementary material for: RPLP2 activates TLR4 in an autocrine manner and promotes HIF-1α-induced metabolic reprogramming in hepatocellular carcinoma
Source: Cell Death Discov. 2023 Dec 5;9:440. doi: 10.1038/s41420-023-01719-0 (PMC10697958; doi:10.1038/s41420-023-01719-0)
Supplement: Supplementary file 1 — WB Original image [file 41420_2023_1719_MOESM1_ESM.pdf]

Fig.1 D

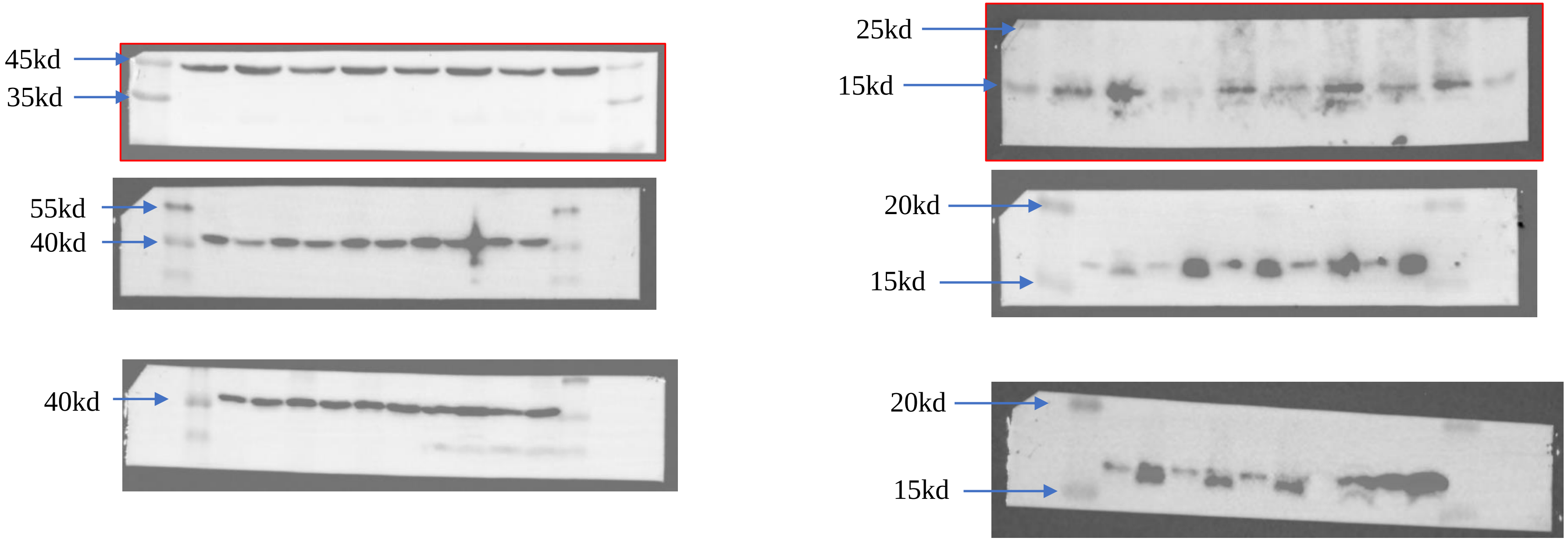

actin

RPLP2

Fig.1 G

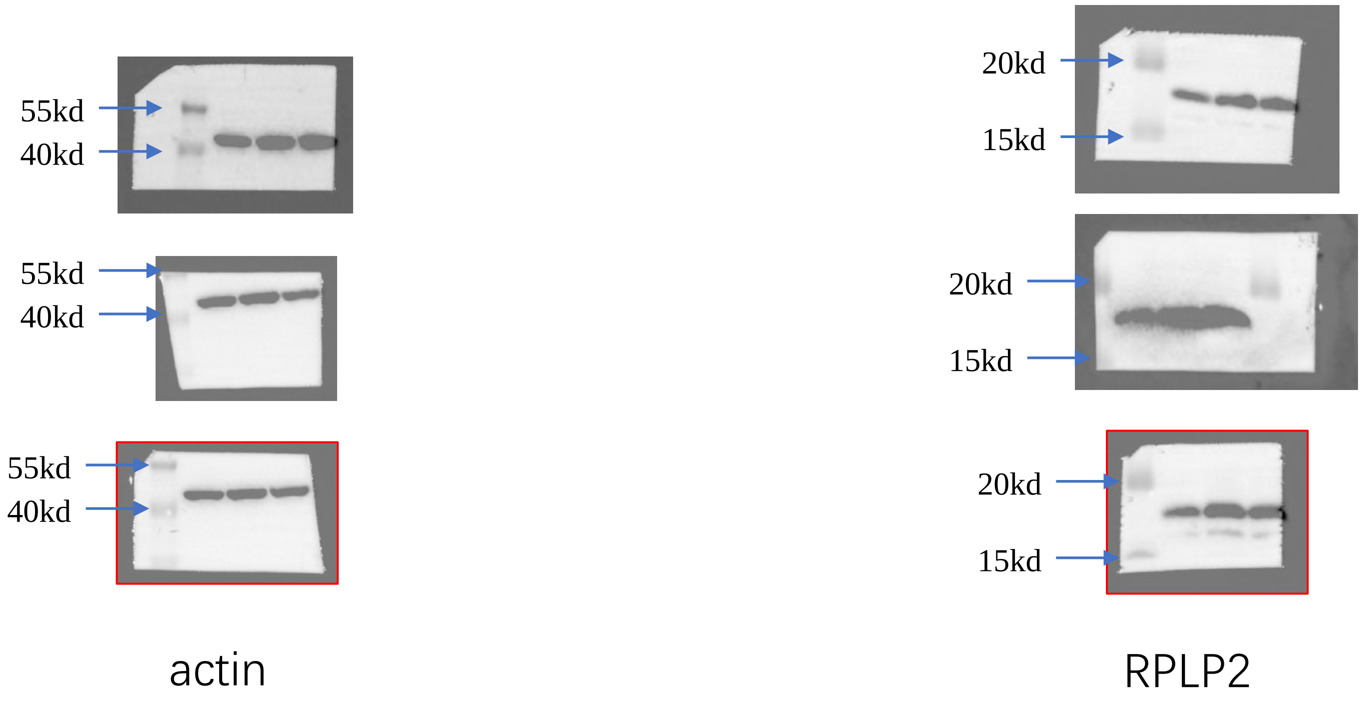

55kd
40kd
55kd
40kd
55kd
40kd
actin
20kd
15kd
20kd
15kd
20kd
15kd
RPLP2

Fig.2 A

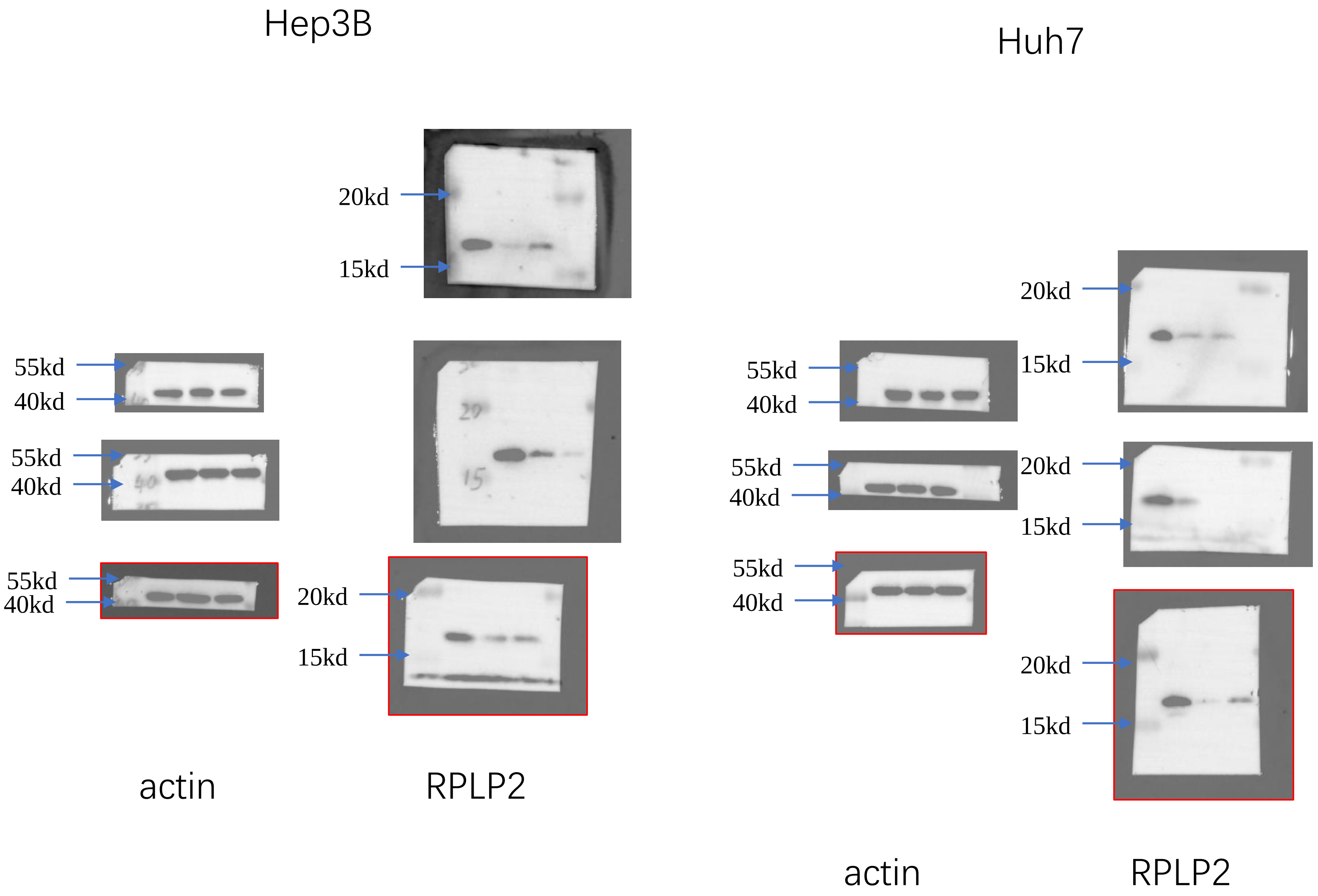
Hep3B
20kd
15kd
55kd
40kd
55kd
40kd
55kd
40kd
20kd
15kd
actin
RPLP2
Huh7
20kd
15kd
55kd
40kd
55kd
40kd
20kd
15kd
55kd
40kd
20kd
15kd
actin
RPLP2

Fig.3 B Hep3B

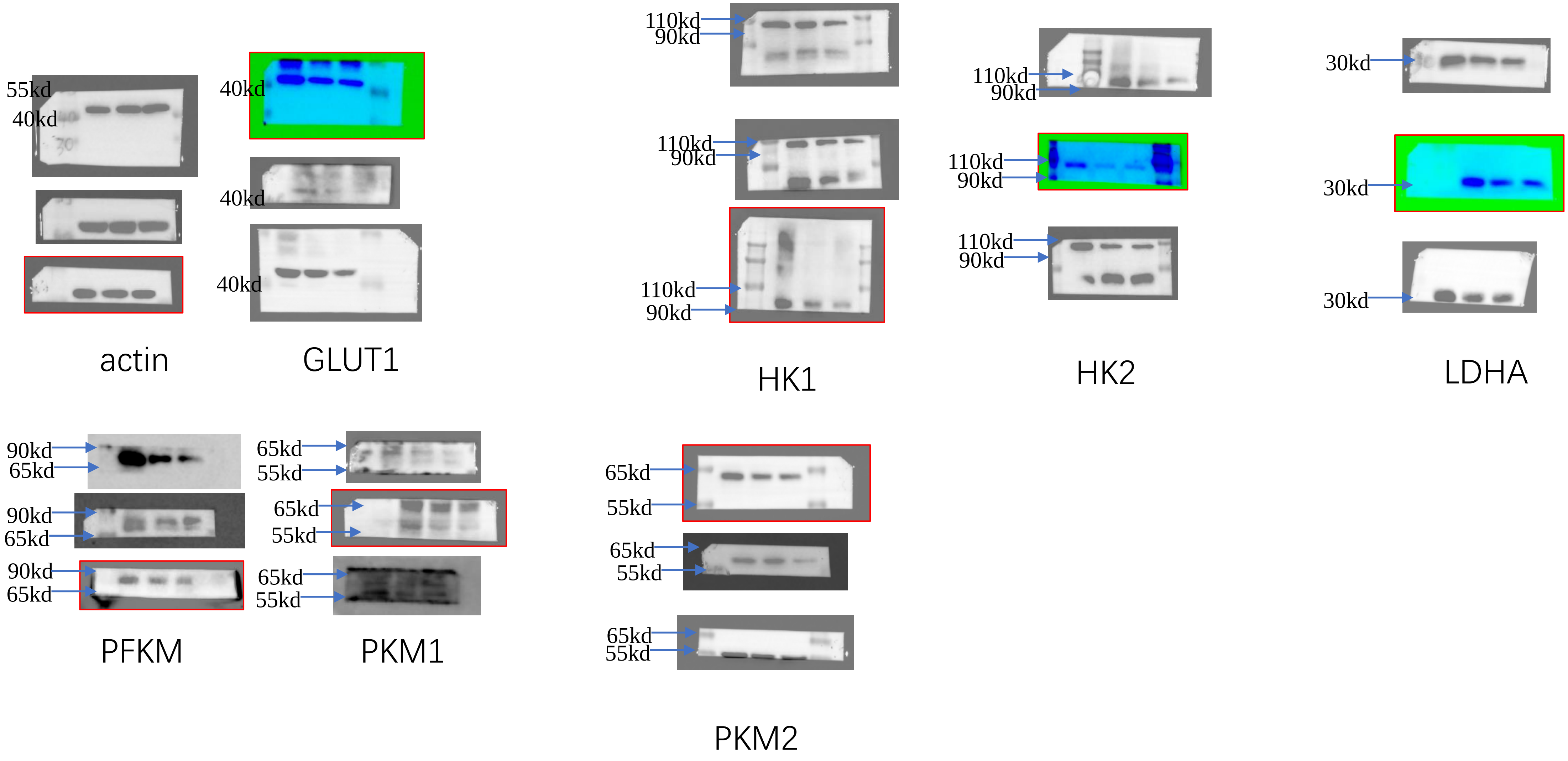
55kd
40kd
40kd
40kd
40kd
actin
GLUT1
110kd
90kd
110kd
90kd
110kd
90kd
HK1
110kd
90kd
110kd
90kd
110kd
90kd
HK2
30kd
30kd
30kd
LDHA
90kd
65kd
90kd
65kd
90kd
65kd
PFKM
65kd
55kd
65kd
55kd
65kd
55kd
PKM1
65kd
55kd
65kd
55kd
65kd
55kd
PKM2

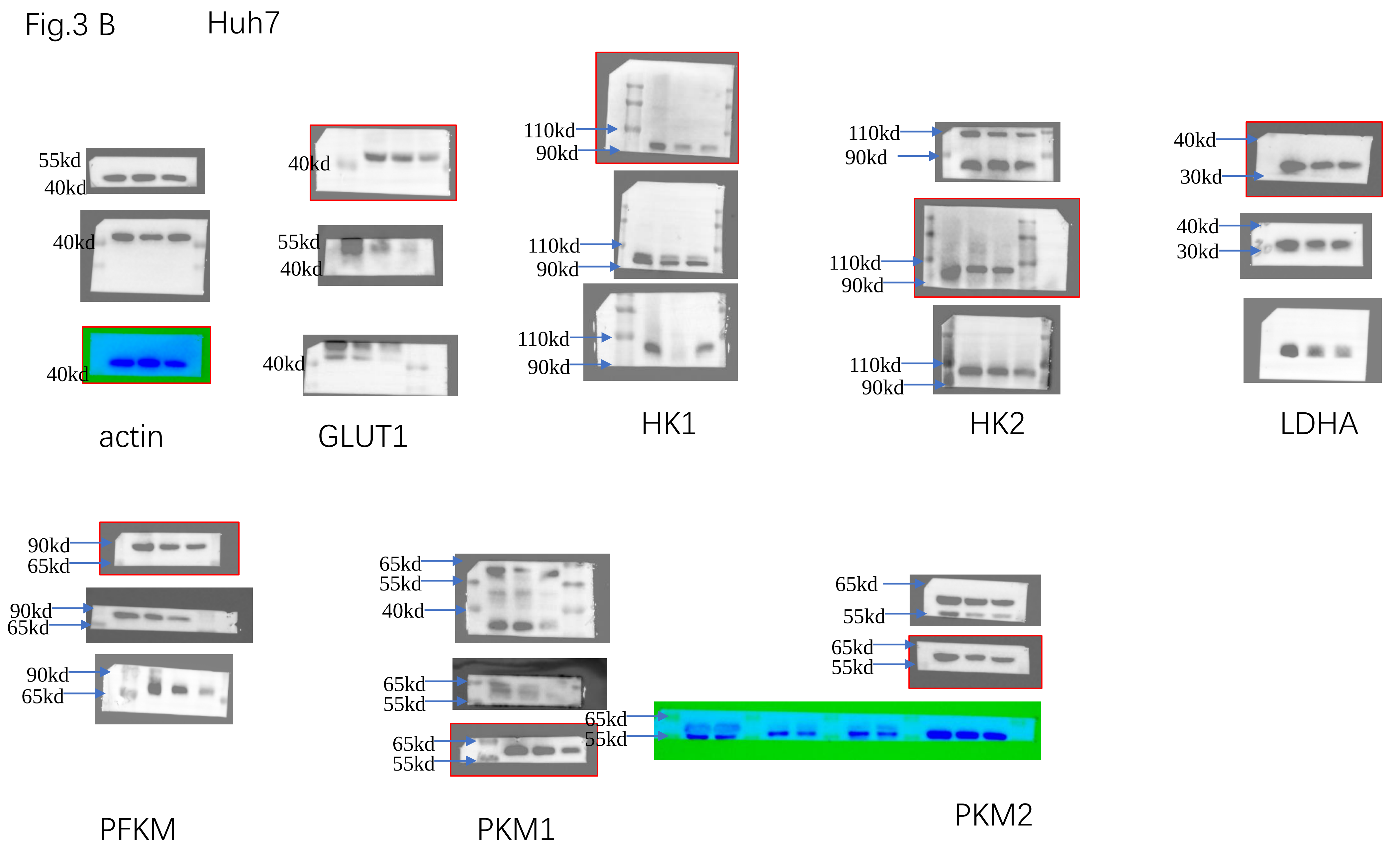
Fig.3 B
Huh7
55kd
40kd
40kd
40kd
actin
40kd
55kd
40kd
40kd
GLUT1
110kd
90kd
110kd
90kd
110kd
90kd
HK1
110kd
90kd
110kd
90kd
110kd
90kd
HK2
40kd
30kd
40kd
30kd
LDHA
90kd
65kd
90kd
65kd
90kd
65kd
PFKM
65kd
55kd
40kd
65kd
55kd
65kd
55kd
PKM1
65kd
55kd
65kd
55kd
65kd
55kd
PKM2

Fig.3 E

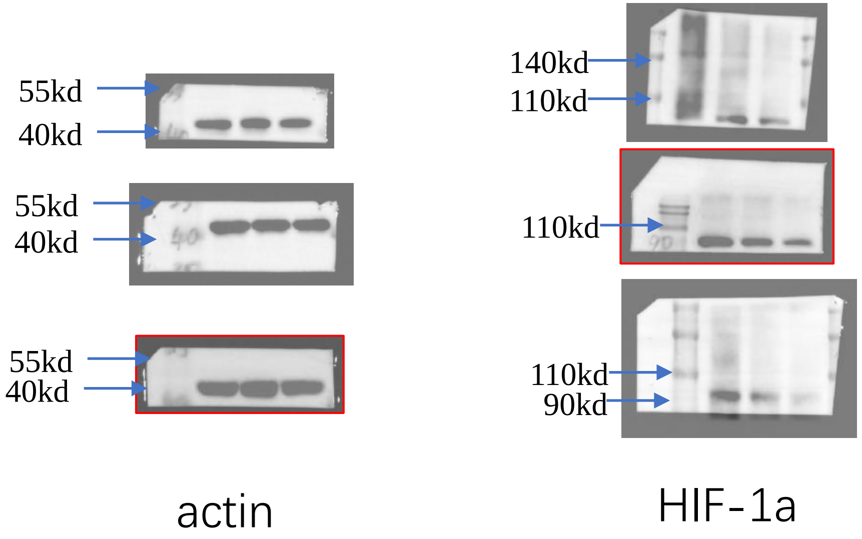
55kd
40kd
55kd
40kd
55kd
40kd
140kd
110kd
110kd
110kd
90kd
actin
HIF-1a

Fig.3 G

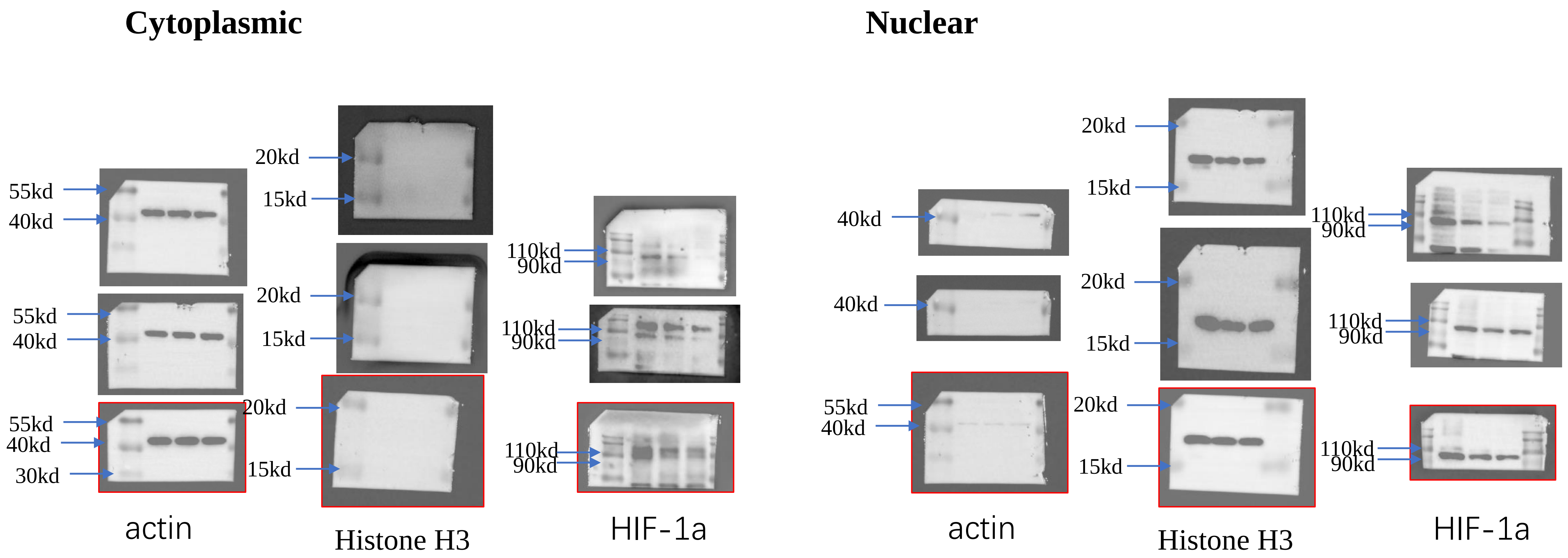

Cytoplasmic
Nuclear
55kd
40kd
30kd
20kd
15kd
110kd
90kd
actin
Histone H3
HIF-1a

Fig.4 A

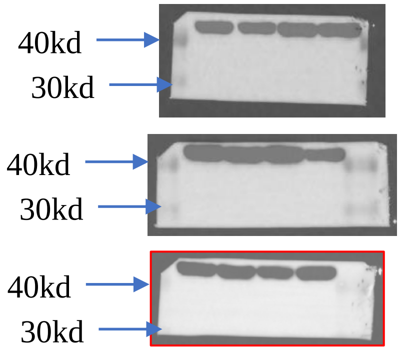

actin

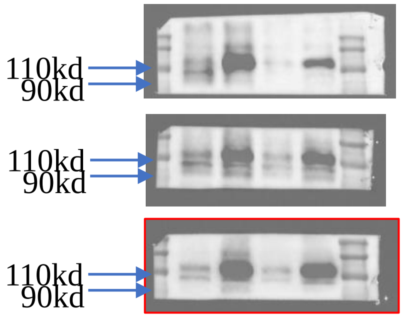

HIF-1a

Fig.4 H

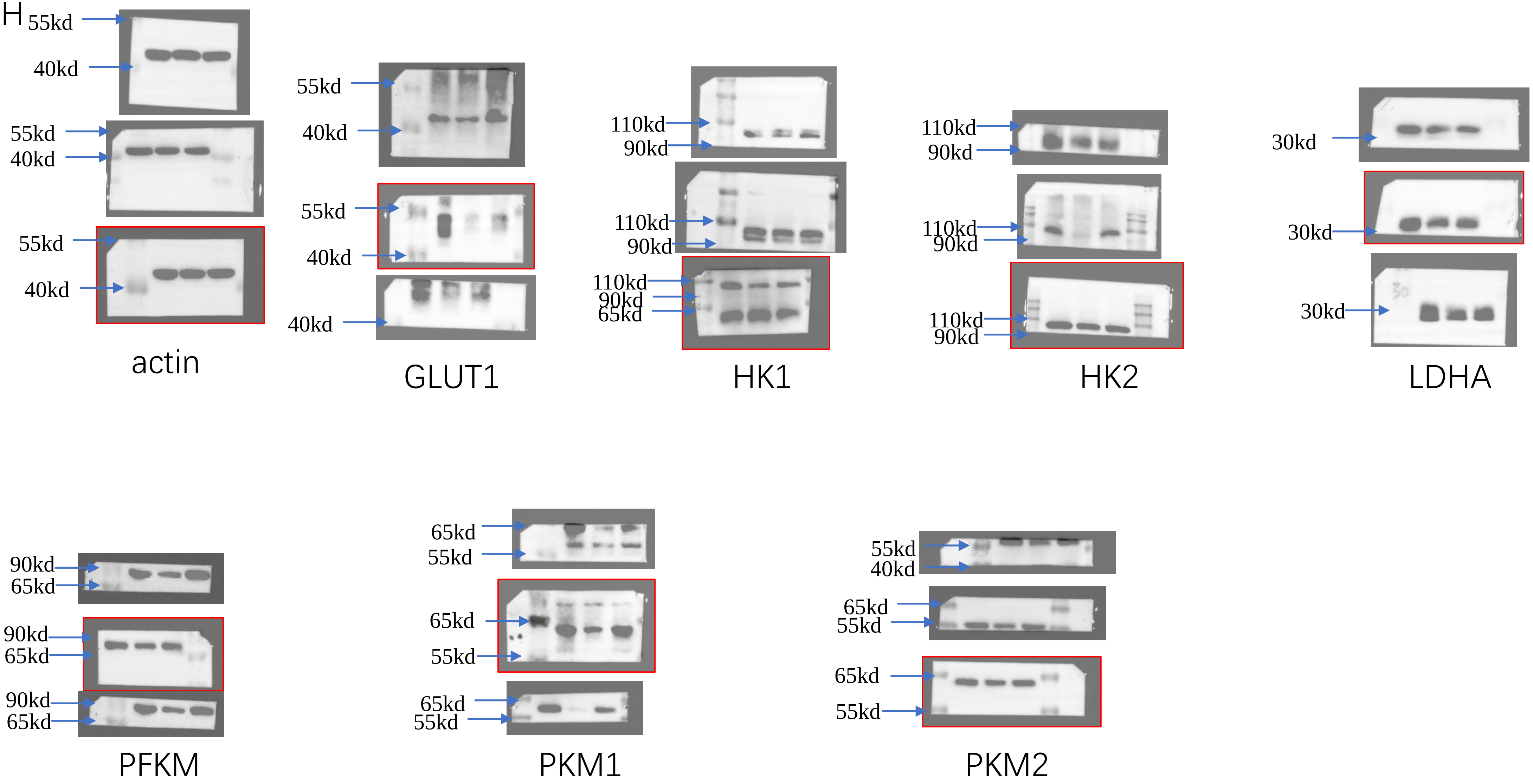
55kd
40kd
55kd
40kd
55kd
40kd
actin
55kd
40kd
55kd
40kd
40kd
GLUT1
110kd
90kd
110kd
90kd
110kd
90kd
65kd
HK1
110kd
90kd
110kd
90kd
110kd
90kd
HK2
30kd
30kd
30kd
LDHA
90kd
65kd
90kd
65kd
90kd
65kd
PFKM
65kd
55kd
65kd
55kd
65kd
55kd
PKM1
55kd
40kd
65kd
55kd
65kd
55kd
PKM2

Fig.5 B

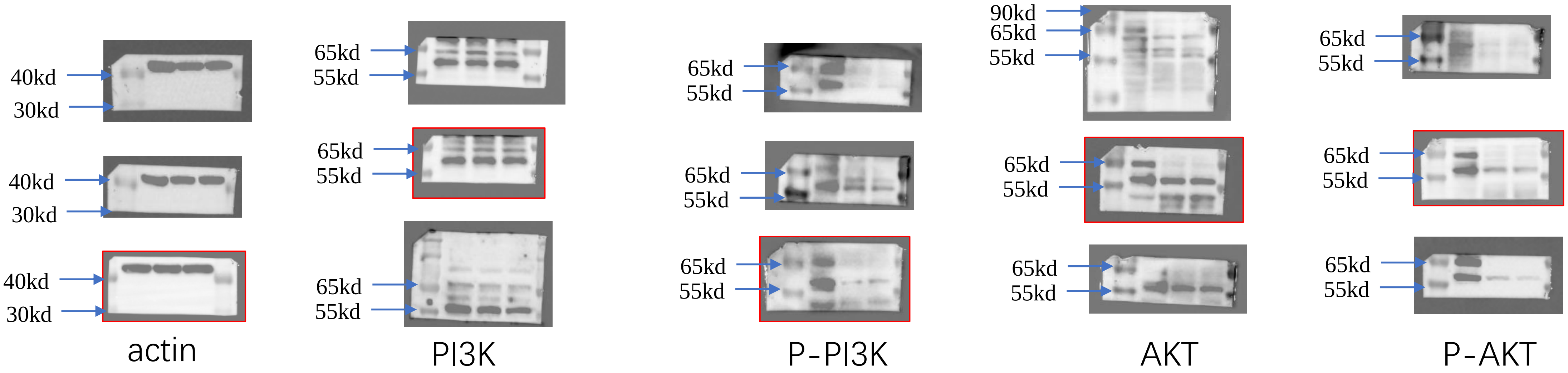
40kd
30kd
40kd
30kd
40kd
30kd
actin
65kd
55kd
65kd
55kd
65kd
55kd
PI3K
65kd
55kd
65kd
55kd
65kd
55kd
P-PI3K
90kd
65kd
55kd
65kd
55kd
65kd
55kd
AKT
65kd
55kd
65kd
55kd
65kd
55kd
P-AKT

Fig.5 D

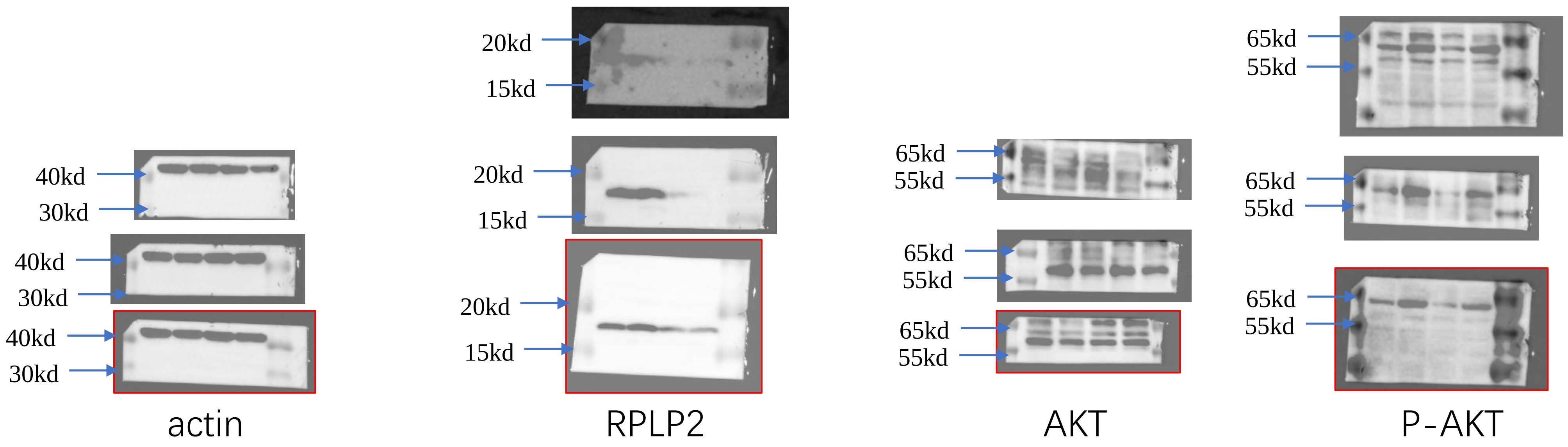
40kd
30kd
40kd
30kd
40kd
30kd
actin
20kd
15kd
20kd
15kd
20kd
15kd
RPLP2
65kd
55kd
65kd
55kd
65kd
55kd
AKT
65kd
55kd
65kd
55kd
65kd
55kd
P-AKT

Fig.5 D

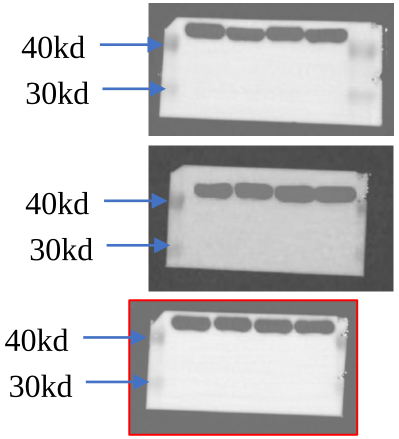

actin

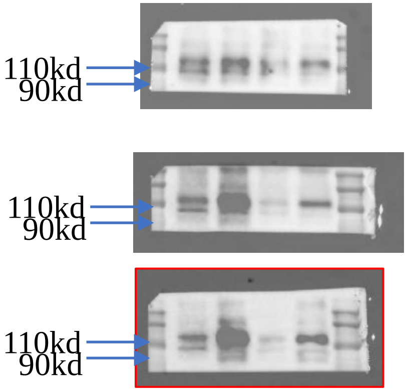

HIF-1a

Fig.5F

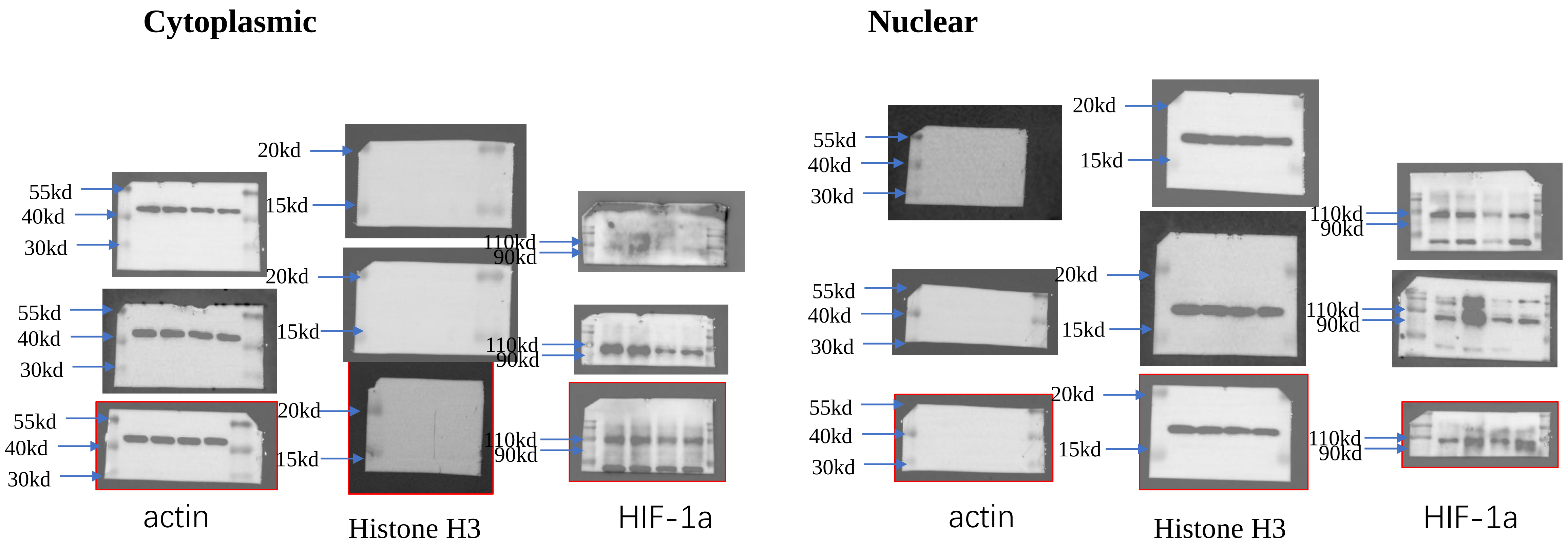
Cytoplasmic
Nuclear
55kd
40kd
30kd
20kd
15kd
110kd
90kd
actin
Histone H3
HIF-1a
actin
Histone H3
HIF-1a

Fig.6 B

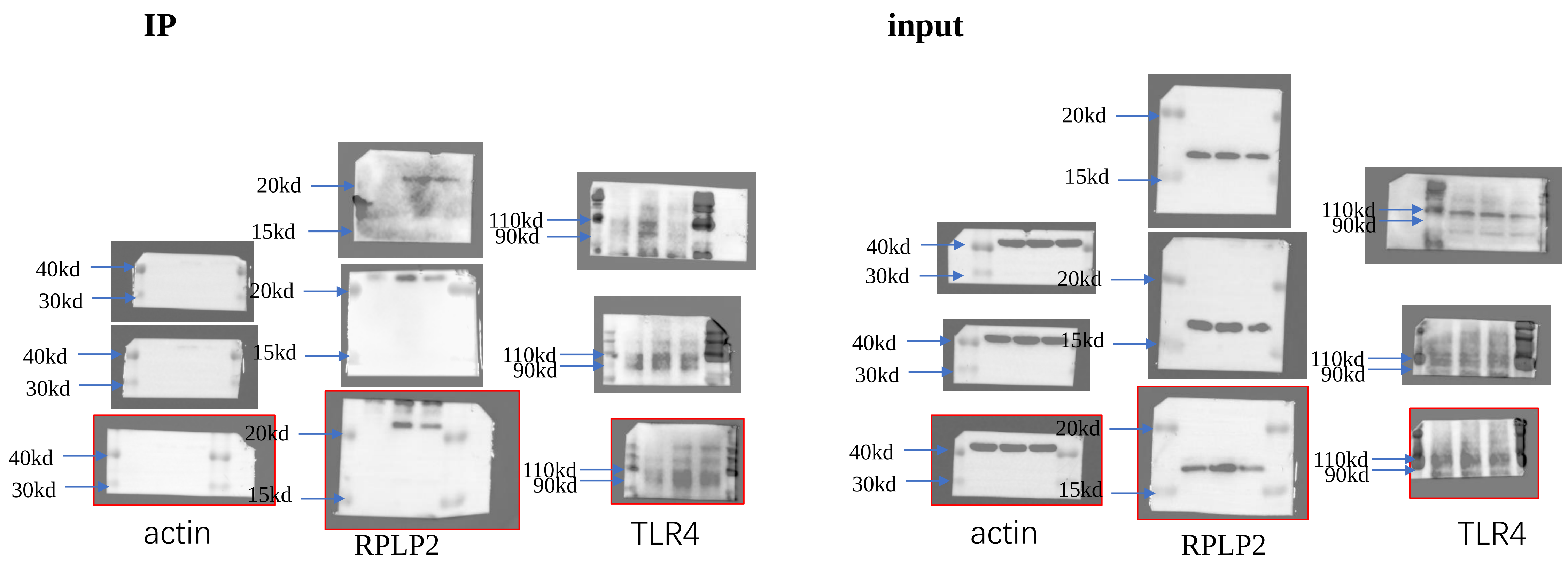

Fig.6 C

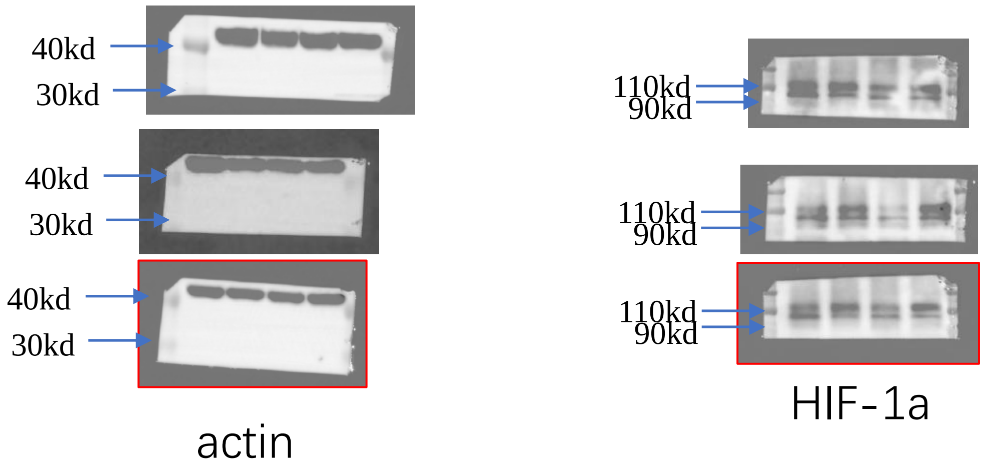
40kd
30kd
40kd
30kd
40kd
30kd
actin
110kd
90kd
110kd
90kd
110kd
90kd
HIF-1a

Fig.6 E

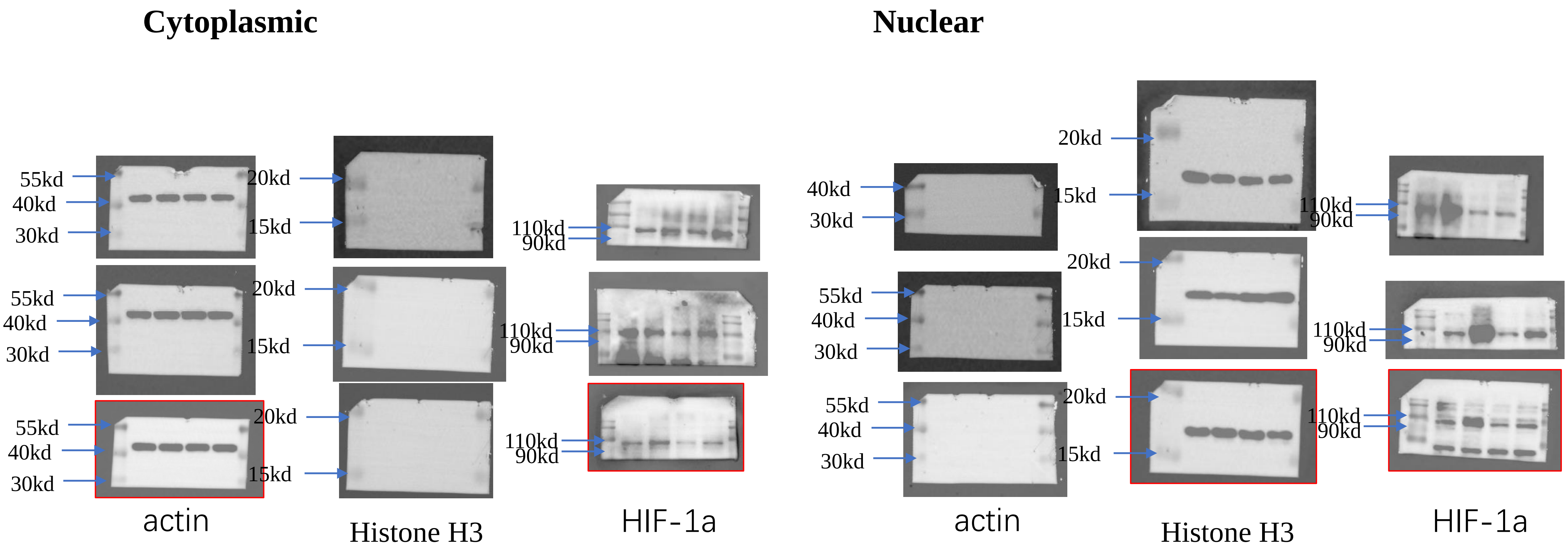
Cytoplasmic
Nuclear
55kd
40kd
30kd
20kd
15kd
110kd
90kd
actin
Histone H3
HIF-1a

Fig.6 H

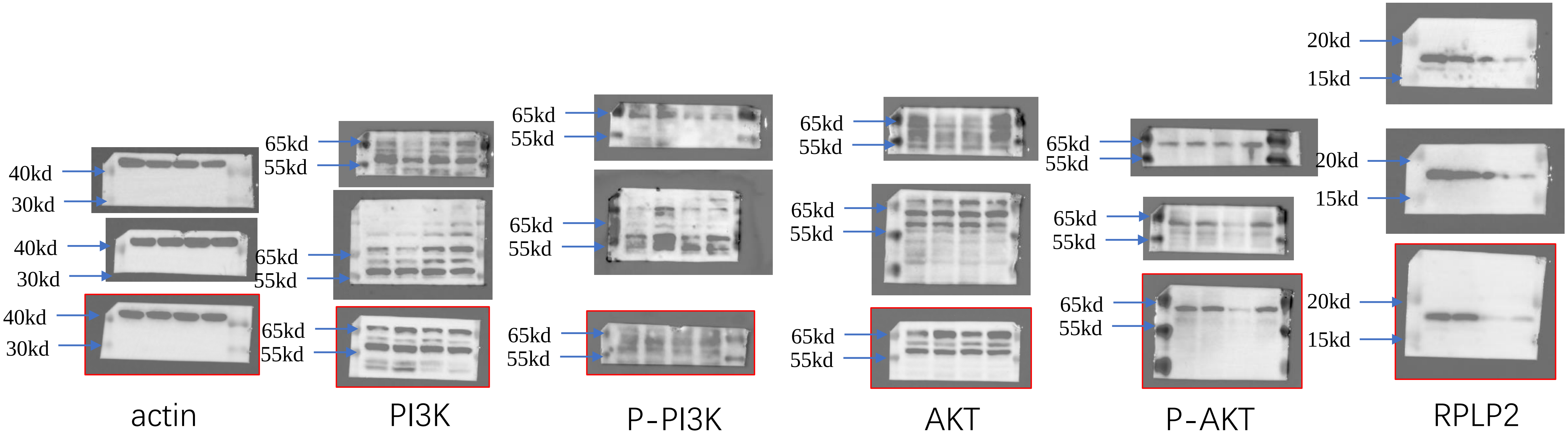
40kd
30kd
65kd
55kd
20kd
15kd
actin
PI3K
P-PI3K
AKT
P-AKT
RPLP2
